# Enhancement of the parent vessel in a giant thrombosed aneurysm

**DOI:** 10.1055/s-0043-1767820

**Published:** 2023-05-31

**Authors:** Flávia Sprenger, Zeferino Demartini Junior, Bernardo Corrêa de Almeida Teixeira

**Affiliations:** 1 Universidade Federal do Paraná, Hospital de Clínicas, Departamento de Radiologia, Curitiba PR, Brazil.; 2 Universidade Federal do Paraná, Hospital de Clínicas, Departamento de Hemodinâmica, Curitiba PR, Brazil.

**Figure 1:**
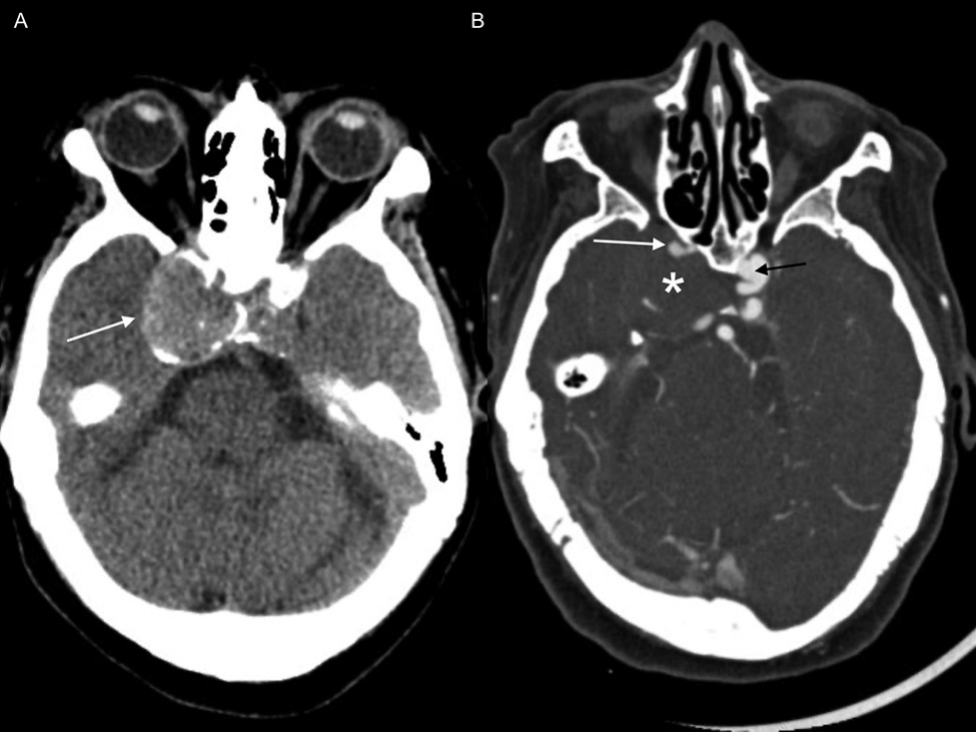
Unenhanced axial computed tomography (CT) scan ( **A** ) and CT angiography ( **B** ) showing a hyperdense mass adjacent to the right cavernous sinus with peripheral calcifications (arrow in **A** ) and no enhancement (asterisk in **B** ), opacification of the pervious internal carotid artery (white arrow in **B** ), and a small contralateral aneurysm (black arrow).

**Figure 2:**
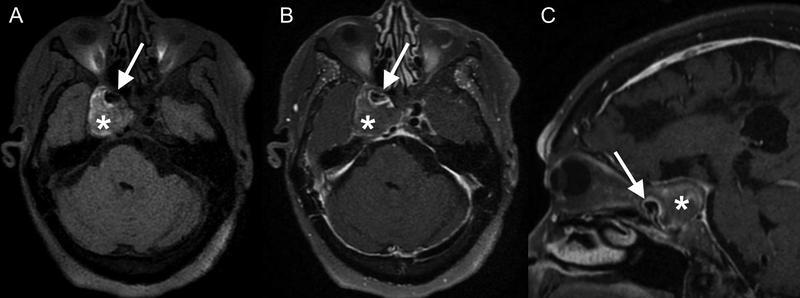
Pre- ( **A** ) and postgadolinium vessel wall magnetic resonance images ( **B-C** ) showing the expected flow void of the parent aneurysm (arrow in **A** ) and the giant non-enhancing thrombosed eccentric component (asterisks). Notice the thickened and enhancing wall of the parent internal carotid artery (arrows in **B** and **C** ).

**Figure 3:**
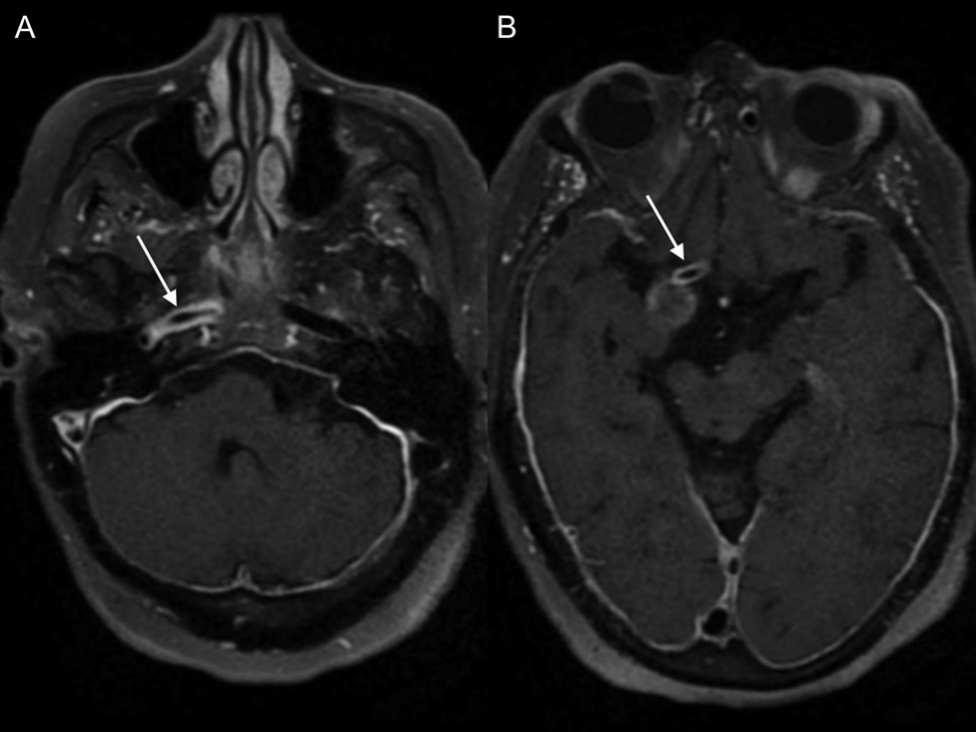
Postgadolinium axial vessel wall magnetic resonance images of the petrous ( **A** ) and remaining precommunicant segments ( **B** ), showing the extension of the arterial wall thickening and enhancement (arrows).

**Figure 4:**
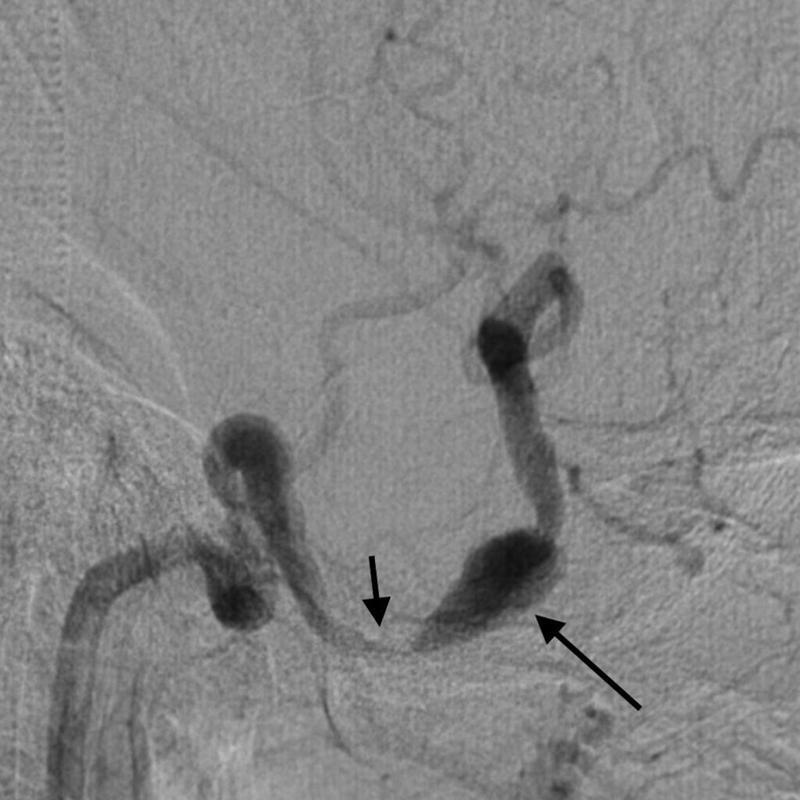
Angiography demonstrating focal stenosis of the right cavernous internal carotid artery due to thrombus compression (short arrow), followed by an aneurysmal dilation (long arrow), with no opacification of the previously-described thrombosed part.

A 62-year-old female with a previous history of 2 ischemic strokes presented with sudden headache and left hemiparesis. Imaging revealed a partially-thrombosed right cavernous carotid artery aneurysm ( Figures 1 and 2 ). Vessel wall imaging showed an extensive vascular wall enhancement of the parent vessel, which might be related to vasa vasorum or inflammation ( Figure 3 ). The role of vascular inflammation within the defective areas of the aneurysm are well known. However, less explored are the inflammatory changes of the parent vessel, which can be detected by magnetic resonance (MR) angiography ( Figure 4 ) and indicate a pathologic artery more subject to aneurysmal formation and thrombotic events. 1 2
